# Identification of genetic determinants of hemolytic activity of *Riemerella anatipestifer* using random transposon mutagenesis

**DOI:** 10.1186/s13567-021-00900-6

**Published:** 2021-02-12

**Authors:** Bingqing Sun, Yafei Xue, Xiaoli Du, Xiaohua He, Zuocheng Zou, Xiangqiang Tian, Zhonghao Hu, Haoyang Liu, Nazrul Islam, Qinghai Hu

**Affiliations:** 1 Shanghai Veterinary Research Institute, the Chinese Academy of Agricultural Sciences, 518 Ziyue Road, Shanghai, 200241 China; 2 Shanghai Animal Disease Control Center, 30 Lane 855 Hongjing Road, Shanghai, 201103 China

**Keywords:** *Riemerella anatipestifer*, Random transposon mutagenesis, Hemolytic activity, Hemolysin, Recombinant protein

## Abstract

*Riemerella anatipestifer* causes epizootic infectious disease in poultry resulting in serious economic losses especially to the duck industry. In our previous study, *R. anatipestifer* was found to lyse duck erythrocytes in vitro. In the present study, a random Tn4351 mutagenesis library of hemolytic *R. anatipestifer* strain SX containing 4000 mutants was constructed to investigate the genetic basis of hemolytic activity. Thirty mutants with reduced hemolytic activity and one with increased hemolytic activity were screened and insertions in 24 genes were identified. Of these genes, four were predicted to encode outer membrane proteins, one encoded a cytoplasmic membrane protein, 11 encoded cytoplasmic proteins, and eight encoded proteins with unknown locations. Based on current annotations of the *R. anatipestifer* genomes, of the 24 genes, 7 (29.17%) were involved in iron utilization. The hemolytic activities of the complemented strains M2 (pRES-Riean_0790) and M18 (pRES-Riean_0653) were restored, indicating that both Riean_0653 and Riean_0790 are involved in the hemolytic activity of strain SX. However, the recombinant proteins rRiean_0317, rRiean_0790, rRiean_0653, rRiean_1027, rRiean_1143, and rRiean_1561 had no hemolytic activity, suggesting that none were hemolysins.

## Introduction

*Riemerella anatipestifer*, the type species of the genus *Riemerella* in the family *Flavobacteriaceae*, mainly infects domestic ducks, geese, turkeys, and other birds. *R. anatipestifer* infection primarily causes an acute septicemic disease in younger birds and more chronic and localized lesions in older birds, resulting in significant economic losses to the duck industry worldwide [1]. However, the mechanisms underlying the pathogenesis of *R. anatipestifer* infection remain unclear.

Some *R. anatipestifer* strains caused hemolysis on 5% bovine blood agar over a 14-day examination period [2] and on Columbia agar base with 7% defibrinated sheep blood [3]. A previous study by our group showed that 29 (55.77%) of 52 *R. anatipestifer* strains caused hemolysis on duck blood agar and observations by phase contrast microscopy showed that all of the strains generated pores in the membranes of duck erythrocytes in suspension [4]. Although the virulence of strong hemolytic *R. anatipestifer* strains may not be greater than that of weak hemolytic strains [4], a mutant with enhanced hemolytic activity and increased pathogenicity to ducklings was identified (Hu et al. unpublished data), suggesting that hemolytic activity may be related to virulence in at least one *R*. *anatipestifer* strain. However, in *R. anatipestifer*, with the exception of the *cam* gene, encoding cohemolysin, which confers cohemolytic activity of *Staphylococcus aureus* on trypticase soy agar supplemented with 5% sheep blood (Christie-Atkins-Munch-Peterson test) [5], no genes associated with hemolysis have yet been identified.

Transposon-based random mutagenesis of bacterial genomes is a powerful genetic tool for the identification of genes and regulatory elements that contribute to specific phenotypes. The most frequently used transposons in bacterial genetics are based on the Tn*5*, Tn10, and Himar1 mariner platforms [6]. However, transposons Tn*5* and Himar1 have no or low transposition frequency in *R. anatipestifer* (Hu et al. unpublished data). Transposon Tn4351 has been used for transposon mutagenesis of *Bacteroidetes* sp. [7, 8]. In our previous studies, Tn4351 was successfully used to construct random transposon mutant libraries of *R. anatipestifer*, which included 33 genes involved in biofilm formation in *R. anatipestifer* strain CH3 [9] and 28 potential virulence-associated genes in *R. anatipestifer* strain YZb1 [10].

In the present study, a random Tn4351 transposon mutagenesis library was successfully constructed, which included 24 genes associated with the hemolytic activity of *R. anatipestifer* strain SX.

## Materials and methods

### Bacterial strains and culture conditions

Hemolytic *R. anatipestifer* strain SX was isolated from the brain of a sickened Cherry Valley duck in our laboratory in 2010 [4]. Erythromycin resistance is conferred to strain SX via the *ereD* gene [11]. *Escherichia coli* strain BW19851 carrying the plasmid pEP4351 [12] was a generous gift from Professor Mark J. McBride of the University of Wisconsin-Milwaukee (Milwaukee, WI, USA). *R. anatipestifer* cells were cultured at 37 °C in tryptic soybean broth (Difco Laboratories Inc., Detroit, MI, USA) or on tryptic soy agar, while *E. coli* cells were routinely grown in Luria broth (LB; Difco Laboratories Inc.) or on LB agar at 37 °C. For selective growth of the bacteria, the following antibiotics (concentrations) were added: ampicillin (100 μg/mL), chloramphenicol (34 μg/mL), cefoxitin (0.5 μg/mL), and kanamycin (50 μg/mL). The strains, plasmids, and primers used in this study are listed in Additional file 1.

### Construction of the recombinant plasmid pEP4351-cfxA

The *cfxA* expression box was amplified from plasmid pCP29 by polymerase chain reaction (PCR) and ligated into the pGEM-T easy vector (Promega Corporation, Madison, WI, USA), which generated T-cfxA. Then, the *cfxA* gene was deleted from T-cfxA with the endonucleases *PpuM*I and *Afl*II, and ligated into the plasmid pEP4351 to generate the plasmid pEP4351-cfxA, in which parts of the *ermF* and *tetX* genes in transposon Tn4351 DNA were replaced with the *cfxA* expression box.

### Generation of a mutagenesis library of *R. anatipestifer* strain SX Tn4351

Tn4351 insertion mutagenesis was performed as described previously [10] with *E. coli* strain BW19851 (pEP4351-cfxA) as the donor and *R. anatipestifer* strain SX as the recipient. After conjugation, the plasmid pEP4351 from *E. coli* BW19851 was introduced into *R. anatipestifer* strain SX and then transposon Tn4351 was randomly inserted into SX genome. The transconjugants were selected on tryptic soy agar containing cefoxitin and kanamycin with the 16S rRNA^+^cfxA^+^ transconjugant as the positive mutant.

### Screening of mutants with decreased or increased hemolytic activity

The hemolytic activities of the wild-type (WT) strain SX and transposon insertion mutants were determined on duck blood agar plates (LB agar base supplemented with 3.5% duck blood, pH 7.5) as described previously [4]. Briefly, suspended bacterial cells were plated on blood agar plates, which were then incubated at 37 °C under an atmosphere of 5% CO_2_/95% air for 24 h, followed by incubation overnight at 4 °C. The hemolytic zones on duck blood agar generated by the mutants and WT SX strain were compared. Transposon insertion mutants with increased or reduced hemolytic activities were isolated. The hemolytic activities of the mutants were determined at least twice on duck blood agar.

### Identification of transposon insertion sites

The genomic DNA of the transposon insertion mutants with decreased or increased hemolytic activity was extracted using the TIANamp Bacteria DNA Kit (Tiangen Biotech (Beijing) Co., Ltd., Beijing, China). The nucleotide sequences flanking the transposon insertion sites were determined using inverse PCR or genomic walking as described previously [9, 13, 14]. The sequences of the identified genes were used to search for other known homologous sequences and putative functions using the Basic Local Alignment Search Tool (BLAST) server [15] and the online PSORTb v.3.0 program [16] was used to predict the subcellular locations of the proteins.

Southern blot analysis of the Tn4351 insertions was used to identify the mutants. The genomic DNA of the tagged Tn4351 insertion mutants was digested with *Xba*I, separated by gel electrophoresis, and transferred to nylon membranes, essentially as described previously [9]. The transposons were identified by detection of the *cfxA* gene. The DIG DNA Labeling and Detection Kit (Roche Diagnostics USA, Indianapolis, IN, USA) was used to prepare the probes and perform hybridization. The number of *cfxA* bands on the nylon membrane represented the number of transposon insertion sites in one mutant.

### Complementation of the mutant strains

To determine whether the decreased hemolytic phenotype of the mutants was due to an inactivated gene, mutants M2 and M18, in which the Riean_0790 and Riean_0653 genes were inactivated by insertion of transposon Tn4351, respectively, were used for a complementation experiment. The open reading frames of Riean_0653 and Riean_0790 were amplified from the WT SX strain by PCR and subcloned into the *E. coli*–*R. anatipestifer* shuttle plasmid pRES [17] generating pRES-Riean_0653 and pRES-Riean_0790, respectively. Then, the plasmids pRES-Riean_0790 and pRES-Riean_0653 were introduced into the mutants M2 and M18 by conjugation generating the complemented strains M2 (pRES-Riean_0790) and M18 (pRES-Riean_0653), respectively.

### Animal experiments

One-day-old Cherry Valley ducklings were obtained from the Lijia Duck Farm (Wuxi, Jiangsu province, China). Serum samples of the ducklings were free of antibodies against *R. anatipestifer* as detected with an indirect enzyme-linked immunosorbent assay using whole cells of strain SX as the coating antigen. All animal experiments were conducted in strict accordance with the recommendations of the Guide for the Care and Use of Laboratory Animals of Shanghai Veterinary Research Institute, the Chinese Academy of Agricultural Sciences (CAAS), Shanghai, China. The study protocol was approved by the Committee on the Ethics of Animal Experiments of the Shanghai Veterinary Research Institute, CAAS (permit no. SHVRI-SV-20201119–02).

To determine whether the genes inactivated in the mutants that caused defects in hemolytic activity had an influence on virulence, the median lethal dose (LD_50_) values of the WT SX strain, the mutant strains M2 and M18, and the complemented strains M2 (pRES-Riean_0790) and M18 (pRES-Riean_0653) were measured using 8-day-old Cherry Valley ducklings as described previously [18].

### Expression of rRiean_1561, rRiean_1143, rRiean_0790, rRiean_0653 and rRiean_0317 in *E. coli* cells

The open reading frames of Riean_1561, Riean_1143, Riean_0790, Riean_0653, and Riean_0317 from the WT SX strain were amplified, cloned into the expression vector pET30a ( +) (Novagen, Inc., Madison, WI, USA), and then expressed in *E. coli* BL21 (DE3) cells (Novagen, Inc.). The bacteria were collected by centrifugation and lysed by sonication. The recombinant proteins rRiean_0317, rRiean_0653, and rRiean_1143 were expressed in *E. coli* cells mainly in the soluble form, while rRiean_1561 and rRiean_0790 were expressed mainly in inclusion bodies. The soluble recombinant proteins rRiean_0317, rRiean_0653, and rRiean_1143 were purified by Ni-iminodiacetic acid affinity chromatography (Detai Bio-Tech (Nanjing) Co., Ltd., Nanjing, China), and the recombinant fusion proteins rRiean_1561 and rRiean_0790 were obtained after washing and dissolution of the inclusion bodies, purification by affinity chromatography, and refolding as previously described [19]. All purified recombinant proteins were soluble in solution buffer (50 mM Tris [pH 8.0] and 150 mM NaCl) and the concentration of each recombinant protein solution was measured using the BCA Protein Assay kit (Pierce Biotechnology, Waltham, MA, USA). The proteins were separated by sodium dodecyl sulfate polyacrylamide gel electrophoresis and identified by Western blot analysis using anti-His tag antibodies (Beyotime Institute of Biotechnology, Haimen, China).

### Hemolytic activities of the complemented strains and recombinant proteins

The hemolytic activities of the WT SX strain, mutant strains M2 and M18, and complemented strains were measured on duck blood agar as described previously [4], and the hemolytic zones generated by these strains were observed. The hemolytic activities of the recombinant proteins rRiean_0317, rRiean_0790, rRiean_1027 (rOmpA1467) [19], rRiean_0653, rRiean_1143, and rRiean_1561 were also monitored on duck blood agar as described previously [4]. The solution buffer was used as a negative control. In addition, to further determine whether these recombinant proteins could perforate the duck erythrocyte membrane, 2% (v/v, in Williams’ Medium E; Sigma-Aldrich Corporation, St. Louis, MO, USA) duck red blood cells were mixed with each recombinant protein in the wells of a 96-well round-bottomed cell culture plate (Corning Incorporated, Corning, NY, USA) and incubated for 12 h at 37 °C. Duck blood cells alone in Williams’ Medium E were used as a negative control. The pelleted non-lysed duck blood cells were washed twice with Williams’ Medium E and the morphologies of the cells were observed under a phase contrast microscope (Eclipse Ci; Nikon Corporation, Tokyo, Japan).

## Results

### Screening of the hemolytic activities of the *R. anatipestifer* SX mutant strains

**Figure 1:**
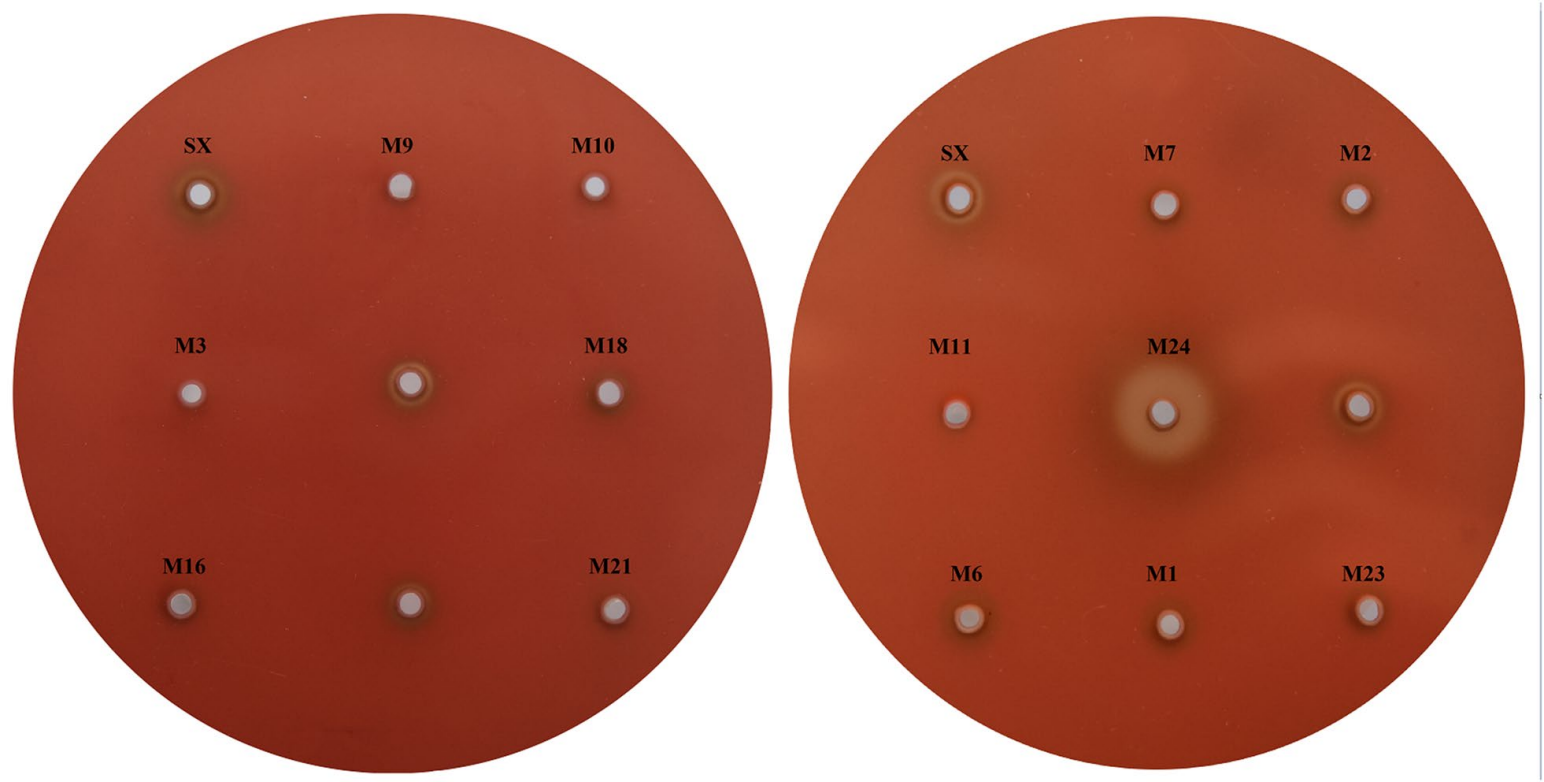
**Hemolytic activities of some of the Tn4351 insertion mutants of*R. anatipestifer*** **strain SX on duck blood agar**. Three mutants with initial defects in hemolytic activity that were not reproducible were not labelled and mutants with mutations in 11 genes (M4, M5, M8, M12, M13, M14, M15, M17, M19, M20, and M22) are not shown.

A random Tn4351 transposon mutagenesis library of *R. anatipestifer* strain SX containing 4000 mutants was constructed with biparental mating of *E. coli* strain BW19851 (pEP4351-cfxA) with *R. anatipestifer* strain SX. The hemolytic activities of all mutants and the WT SX strain were measured on duck blood agar. The mutants that exhibited no or very weak hemolytic zones on duck blood agar were characterized as hemolytic-defective mutants, while those with obvious increased hemolytic zones were characterized as hemolytic-increase mutants (Figure 1). On the basis of these criteria, 30 of the 4000 transposon mutants with no or very weak hemolytic zones and one mutant with an increased hemolytic zone on duck blood agar were identified. These mutants displayed reproducible hemolytic activity on duck blood agar. Southern blot analysis confirmed that each of the 31 mutants had a single Tn4351 insertion.

### Identification of disrupted genes

The GenBank database was searched for genes homologous to the DNA sequences flanking the transposon Tn4351 insertion sites of the 31 mutants. The results of this analysis are shown in Table 1, along with the details of the identified 24 mutated genes. Of these, five genes were found to be inserted by the transposon Tn4351 in different mutants. In mutants M18, M27, M29, and M31, Riean_0653 was inserted by Tn4351 at three sites; in mutants M2 and M25, Riean_0790 was inserted at different sites; in mutants M3 and M26, Riean_1027 was inserted at the same site; in mutants M7 and M28, Riean_0317 was inserted at different sites, and in mutants M21 and M30, Riean_1143 was also inserted at the same site (Additional file 3). The two mutants M3 and M26, in which transposon Tn4351 was inserted at the same site on the genome, were isolated from different experiments, as with M27 and M31, M21 and M30. Therefore, none of the mutants shown in Figure 1 appear to be siblings. In addition, in six mutants (M5, M11, M14, M16, M24 and M28), the transposon Tn4351 was inserted into the putative promoter of the affected gene (Additional file 3). Surprisingly, seven of the 24 genes identified in this study were predicted to be involved in iron utilization based on the current annotations of *R. anatipestifer* genomes.

**Table 1:** Description of hemolytic activity *Riemerella anatipestifer* SX mutants

Mutants	Hemolytic zones^a^	Description of encoded proteins	Locus tag in DSM15868^b^	Gene products		
				Subcellular location^c^	Putative conserved domain	Function group (COGs)^e^
M1	almost no	hypothetical protein	Riean_0373	Outer Membrane	–^d^	–^f^
M2, M25	almost no	hypothetical protein	Riean_0790	Outer membrane	CarboxypepD_reg	–
M3, M26	almost no	OmpA/MotB domain protein	Riean_1027	Outer membrane	OmpA_C_like, OmpA	COG2885 M
M4	almost no	TonB-dependent receptor plug	Riean_1561	Outer membrane	CirA, OMP_RagA_SusC	–
M5	reduced greatly	hypothetical protein	Riean_1374	Cytoplasmic membrane	–	–
M6	almost no	diacylglycerol kinase catalytic region	Riean_0288	Cytoplasmic	LCB5, DAGK_cat, DAGKC	COG1597 IR
M7, M28	almost no	TPR repeat protein	Riean_0317	Cytoplasmic	TPR_1	COG0790 T
M8	reduced greatly	adenylosuccinate lyase	Riean_0391	Cytoplasmic	PurB, ADSL_C, PRK08937	COG0015 F
M9	almost no	Pyruvate dehydrogenase (acetyl-transferring)	Riean_0589	Cytoplasmic	AcoB,Transket_pyr, Transketolase_C TPP_PYR_E1-PDHc-beta_like	COG0022 C
M10	almost no	Mg chelatase, subunit ChlI	Riean_1262	Cytoplasmic	YifB, Mg_chelatase, AAA	COG0606 O
M11	almost no	bacterial translation initiation factor 2	Riean_1302	Cytoplasmic	InfB, IF-2, IF2_Eif5B	COG0532 J
M12	almost no	YicC-like domain-containing protein	Riean_1527	Cytoplasmic	PRK11820, YicC, YicC_N	COG1561 S
M13	almost no	riboflavin biosynthesis protein RibD	Riean_1661	Cytoplasmic	RibD_1_, RibD_C	COG0117 H
M14	almost no	ATPase associated with various cellular activities AAA_3	Riean_1768	Cytoplasmic	AAA_3, AAA	COG0714 R
M15	reduced greatly	RelA/SpoT domain protein	Riean_1808	Cytoplasmic	NT_Rel-Spo_like, RelA_SpoT	COG2357FT
M16	almost no	RagB/SusD domain protein	Riean_0025	Unknown	SusD	-
M17	reduced greatly	alkyl hydroperoxide reductase/thiol specific antioxidant/Mal allergen	Riean_0256	Unknown	Thioredoxin_8, TrxA	COG0450 V
M18, M27, M29, M31	reduced greatly	hypothetical protein	Riean_0653	Unknown	–	–
M19	almost no	outer membrane transport energization protein ExbD1	Riean_0932	Unknown	ExbD, tolR	COG0848 U
M20	almost no	hypothetical protein	Riean_0968	Unknown	–	–
M21, M30	almost no	Zn-dependent aminopeptidase	Riean_1143	Unknown	M1_APN_5	COG2234 O
M22	reduced greatly	PSP1 domain protein	Riean_1185	Unknown	PSP1	COG1774 T
M23	almost no	Ferritin Dps family protein	Riean_1386	Unknown	Dps, Ferritin, PRK09448	COG0783 PV
M24	increased	homogentisate 12-dioxygenase	Riean_1546	Cytoplasmic	HmgA, PLN02658	COG3508 Q

^a^The sizes of hemolytic zone of the mutants on duck blood agar were compared to that of the wild strain SX. The mutants that exhibited almost no, very weak or increased hemolytic zones were examined

^b^GenBank accession No.: CP002346

^c^Subcellular locations were predicted by the PSORTb v.3.0 server [16]

^d^No conserved domain was predicted by Conserved Domain Database (CDD) [24]

^e^COG functional categories: (1) Information storage and processing (J: Translation, ribosomal structure and biogenesis); (2) Cellular processes.(M: Cell wall/membrane/envelope biogenesis; O: Post-translational modification, protein turnover, chaperones; T: Signal transduction mechanisms; V: Defense mechanisms; U: Intracellular trafficking,secretion,and vesicular transport); (3) Metabolism: (C:energy production and vonversion;E: Amino acid transport and metabolism; F: Nucleotide transport and metabolism; P: Inorganic ion transport and metabolism; I: lipid metabolism; G: Carbohydrate transport and metabolism; H. Coenzyme transport and metabolism; Q: secondary metabolites biosynthesis,transport and catabolism;). (4) Poorly characterized: (R: General function prediction only; S: function unknown)

^f^ – No related COG

The subcellular locations of the 24 protein-encoding genes were predicted using PSORTb software [16]. Of these 24 genes, four were predicted to encode outer membrane proteins (OMPs), one encoded a cytoplasmic membrane protein, 11 encoded cytoplasmic proteins, and eight encoded proteins with unknown locations (Table 1). These proteins were further categorized based on putative functions. Of the 24 proteins, nine were classified into the cellular processes and signaling category (M, T, O, U, V. and P), six into the metabolism category (C, F, H, I, and Q), one into the information storage and processing category (J), two as poorly characterized clusters of orthologous groups (R), and seven could not be categorized.

### Hemolytic activities of the mutants M2 and M18 were restored by plasmid complementation

**Figure 2:**
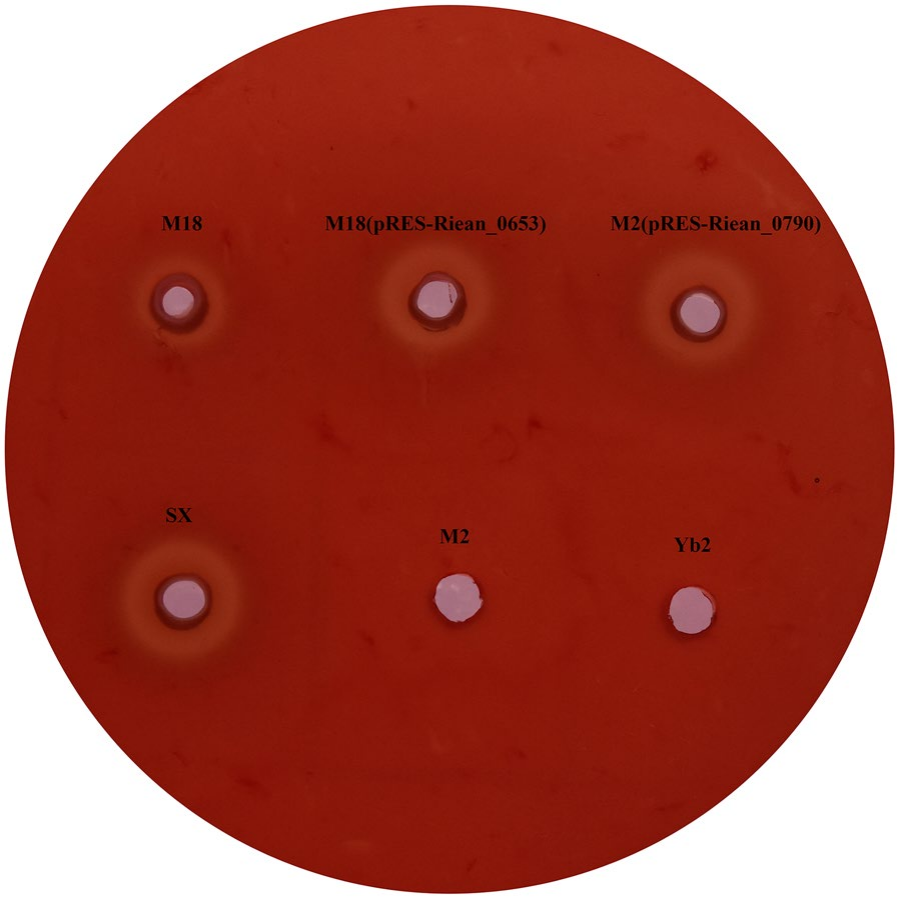
**Hemolytic activity of the mutants M2 and M18 were restored by plasmid complementation**. The WT SX strain, the mutant strains M2 and M18, and the complemented strains M2 (pRES-Riean_0790) and M18 (pRES-Riean_0653) were grown in TSB to the mid-logarithmic phase. The bacterial cell pellets were resuspended in phosphate-buffered saline and 10 μL of the cell suspensions were plated on blood agar. The hemolytic zones generated by these strains were observed. Yb2 (dba^−^ strain) was used as a negative control.

As shown in Figure 2, on duck blood agar, there was no or very weak hemolytic activities of the mutants M2 and M18, while the hemolytic activities of the complemented strains M2 (pRES-Riean_0790) and M18 (pRES-Riean_0653) were almost completely restored to that of the WT SX strain, suggesting that both Riean_0653 and Riean_0790 are involved in the hemolytic activities of *R. anatipestifer* strain SX.

### Determination of the virulence of mutants M2 and M18

**Figure 3:**
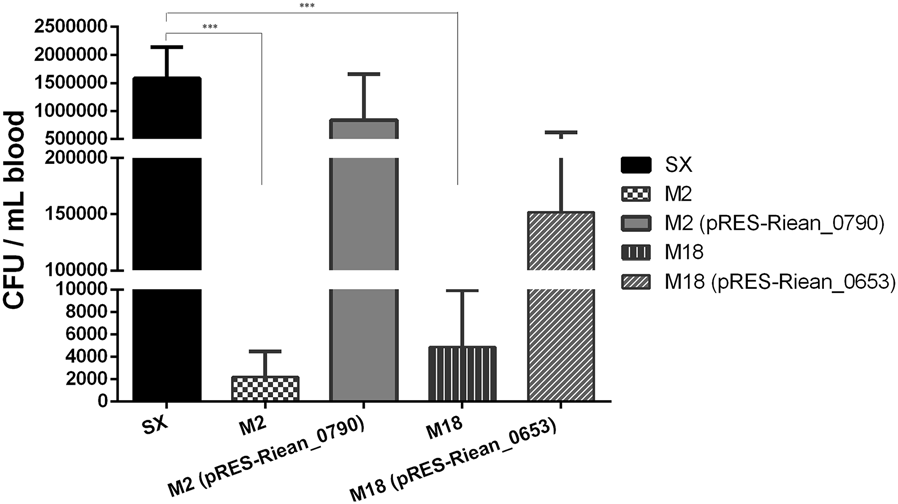
**Blood bacterial loads in ducklings infected with the WT SX, M2, M18, and complemented strains.** The bacterial loads in blood of ducklings infected with the WT SX, M2, M18, M2 (pRES-Riean_0790), and M18 (pRES-Riean_0653) strains were measured at 24 h post-inoculation. The results showed that the bacterial loads of blood of ducklings infected with the complemented strain M2 (pRES-Riean_0790) or M18 (pRES-Riean_0653) were significantly restored as compared to those infected with M2 or M18. The error bars represent means ± standard deviations of eight ducklings. Asterisks indicate statistically significant differences between two groups (***p < 0.0001).

Eight-day old Cherry Valley ducklings were infected with the WT SX strain, mutant strains M2 and M18, and the complemented strains to determine the effect of the mutations on the virulence of the bacterium. The results showed that the LD_50_ values of the WT SX, M2, M18, M2 (pRES-Riean_0790), and M18 (pRES-Riean_0653) were 4.48 × 10^8^, 5.46 × 10^9^, 5.97 × 10^9^, 3.99 × 10^9^, and 4.64 × 10^9^ CFU, respectively. The LD_50_ values of the mutants M2 and M18 were about 12 to 13 fold greater than that of the WT SX strain, suggesting that disruption of Riean_0790 or Riean_0653 may result in attenuation of the virulence of *R. anatipestifer*. The LD50 of the mutants M2 and M18 complemented with the shuttle expression plasmid pRES-Riean_0790 and pRES-Riean_0653 respectively, was not clearly different from that of the non-complemented mutant strains. In addition, as shown in Figure 3, the bacterial loads in the blood of ducklings infected with the mutant strain M2 or M18 were decreased significantly as compared to those infected with the WT SX strain at 24 h post-inoculation (*p* < 0.0001), and the bacterial loads in the blood of ducklings infected with the complemented strain M2 (pRES-Riean_0790) or M18 (pRES-Riean_0653) were significantly restored (*p* < 0.0001), consistent with the restored hemolytic activities of the two complemented strains. These results indicate that the hemolytic activities of these strains might not be directly related to virulence.

### None of the recombinant proteins exhibited hemolytic activity

The results of our previous study showed that both the culture supernatants and OMPs of *R. anatipestifer* had hemolytic activities, indicating that the culture supernatants and OMPs may contain hemolysins [4]. To determine whether the identified genes encoded hemolysins, six genes predicted to encode OMPs or cytoplasmic proteins, especially those inserted in different mutants by transposon Tn4351, were expressed in *E. coli* cells, purified (Additional file 2), and the hemolytic activities were measured. The results showed that all of the tested recombinant proteins (rOmpA1467 (rRiean_1027), rRiean_1561, rRiean_1143, rRiean_0790, rRiean_0653, and rRiean_0317) had no hemolytic activity on duck blood agar. In addition, these six recombinant proteins did not generate pores in the duck erythrocyte membrane (Additional file 4), suggesting that none were hemolysins.

## Discussion

In the present study, a total of 24 genes were identified and 31 mutants with reduced and one with increased hemolytic activity were screened by random Tn4351 transposon mutagenesis using *R. anatipestifer* strain SX as the parent strain. These genes or the encoded proteins may be directly or indirectly involved in the hemolytic activity of *R. anatipestifer*. The encoded proteins may act as hemolysin/cytotoxin molecules to directly lyse duck red blood cells. More likely, these genes or the encoded proteins may indirectly lyse the membranes of duck erythrocytes by (1) the production of hemolysin [20], (2) the export or secretion of hemolysin, (3) acting as a regulator of hemolytic activity; or (4) are other hemolysis-related proteins [21]. However, the exact roles of the genes identified in the present study or the encoded proteins conferring hemolytic activity to *R. anatipestifer* remain to be further confirmed.

The ability of pathogenic bacteria to acquire iron in an animal host cell is a critical determinant for the establishment of infection. Increased levels of hemolytic activity and hemolysins were detected in the supernatants of iron-limited *Vibrio cholerae* cells [22]. *R. anatipestifer* has been reported to acquire iron and/or other nutrients from lysed erythrocytes to promote growth and proliferation both in vitro and in vivo [4]. Seven of the 24 genes identified in this study were predicted to be involved in iron utilization, suggesting that the ability of *R. anatipestifer* to acquire iron is related to its hemolytic activity.

In the mutant M4, Tn4351 was inserted into Riean_1561, which encodes a TonB-dependent receptor plug. TonB-dependent receptors of some bacteria are involved in iron acquisition and host infection. The TonB-dependent receptor TbdR1 of *R. anatipestifer* strain CH3 is involved in hemin iron acquisition and necessary for optimal bacterial virulence [23]. According to the Conserved Domain Database [24], the conserved domain Cir (COG1629) of Riean_1561 is predicted to be mostly involved in iron transport, while OMP_RagA_SusC may be involved in iron utilization of *R. anatipestifer* strain SX.

In the mutant M10, Riean_1262 (yifB gene), which encodes a Mg chelatase, was inserted with the transposon Tn4351, which resulted in significantly decreased hemolytic activity as compared to that of the WT SX strain. However, the exact underlying mechanism involved in this process remains unclear. In *Bacteroides fragilis* lacking the *yifB* gene, ferrochelatase activity was significantly increased by approximately 2.2-fold as compared with the parent strain. In addition, the ability of *B. fragilis* to utilize heme or protoporphyrin IX for growth was greatly reduced in a Δ*uroS* mutant and this Δ*uroS* suppressive effect was enhanced by deletion of the *yifB* gene [25].

In the mutant M13, Tn4351 insertion in Riean_1661 resulted in significantly decreased hemolytic activity. Riean_1661 encodes the riboflavin biosynthesis protein RibD. In bacteria, the expression of genes involved in riboflavin biosynthesis is influenced by iron availability. Riboflavin is involved in overcoming iron-restrictive conditions in some species, which is probably achieved through increased iron bioavailability by reduction of extracellular iron, activities of iron uptake pathways, and hemolytic activity [26].

In the mutant M15, Tn4351 was inserted in Riean_1808, which encoded the RelA/ SpoT domain protein. In *E. coli*, RelA produces pppGpp (or ppGpp) from adenosine triphosphate and guanosine triphosphate, or guanosine diphosphate, and SpoT degrades ppGpp, but may also act as a secondary ppGpp synthetase. Iron was found to be involved in the regulation of the two activities of SpoT: synthesis and hydrolysis of (p)ppGpp. Iron limitation causes SpoT-dependent accumulation of ppGpp, which in turn stimulates the iron uptake capacity of the cell [27].

Tn4351 insertion in Riean_0932 in the mutant M19 led to defective hemolytic activity. ExbD1 encoded by Riean_0932, which is part of the TonB/ExbB/ExbD complex, is required for high-affinity iron transport in Gram-negative bacteria. The results of our previous study showed that two TonB/ExbB/ExbD systems of *R. anatipestifer* are involved in iron acquisition by hemin, hemoglobin, and holo-transferrin, and are necessary for optimal bacterial virulence [22].

In the mutant M23, Tn4351 was inserted in Riean_1386, which encodes a ferritin Dps family protein. Ferritin is the primary iron storage protein and is critical for iron homeostasis. Ferritin makes iron available for critical cellular processes, while protecting lipids, DNA, and proteins from the potentially toxic effects of iron [28]. Tn4351 insertion in Riean_1386 in the mutant M23 may lead to disordered iron storage and homeostasis.

In the mutant M24, transposon Tn4351 insertion in Riean_1546 led to an increase in hemolytic activity. Riean_1546 (*hmgA* gene) encodes homogentisate 1,2-dioxygenase, which can degrade homogentisic acid (HGA). In the absence of HmgA (*hmgA* mutant), HGA accumulates and oxidizes to form of HGA-melanin, and the HGA-melanin pigment secreted by *Legionella pneumophila* was found to confer ferric reductase activity, resulting in excessive reduction of iron [29, 30]. In the present study, Tn4351 insertion in *R. anatipestifer* Riean_1546 may also lead to excessive reduction of iron, and the resulting iron deficiency may upregulate the expression of iron uptake-related genes, including hemolysin, and hemolytic activity, etc. The exact mechanism of negative regulation of Riean_1546 deletion on increased hemolytic activity of *R. anatipestifer* SX will be investigated in a future study.

In addition, in the present study, six recombinant proteins, including rRiean_1027, rRiean_1561, rRiean_1143, rRiean_0790, rRiean_0653, and rRiean_0317, showed no hemolytic activity, indicating that these proteins were not hemolysins. Whether one or more other proteins identified in this study were hemolysins remains unclear, but two predicted hemolysins (Riean_0620 and Riean_0415), according to the genomic annotations of the type strain ATCC11845 [31], were not identified, which may due to the insufficiency of the random transposon mutagenesis library used in this study. Moreover, it seems that transposon Tn4351 preferentially inserts into “TA” nucleotide sequences within the genome of *R. anatipestifer*, which led to the same Tn4351 insertion sites of the genomes of different mutants, as shown in this study. In addition, insertion of a transposon within an operon could result in transcription polarity of the downstream genes. Therefore, although random transposon mutagenesis is a useful tool to identify new functional genes in bacteria, this technology has its limitations.

In the present study, 24 genes were involved in the hemolytic activity of *R. anatipestifer* strain SX and seven were predicted to be related to iron utilization. These results will be helpful to clarify the molecular mechanism underlying the hemolytic activity of *R. anatipestifer*.

## Supplementary Information

**Additional file 1:** **Strains, plasmids and primers used in this study**.

**Additional file 2:** **Detection of purified recombinant proteins by SDS-PAGE and Western blot analysis**. After purification, the recombinant proteins were separated by 12% SDS-PAGE under reducing conditions. Gels were either stained with Coomassie blue or electroblotted onto a polyvinylidene fluoride membrane. Mouse anti-His tag antibody was used as the primary antibody for Western blot analysis. M1: protein marker for SDS-PAGE. M2: protein marker for Western blot analysis. (A) Detection of purified rRiean_0317 by SDS-PAGE and Western blot analysis. (B) Detection of purified rRiean_0373 by SDS-PAGE and Western blot analysis. (C) Detection of purified rRiean_0653 by SDS-PAGE and Western blot analysis. (D) Detection of purified rRiean_1143 by SDS-PAGE and Western blot analysis. (E) Detection of purified rRiean_1561 by SDS-PAGE and Western blot analysis.

**Additional file 3:** **The Location of Tn4351 insertions on the genome of hemolytic-defective mutants.** Since there is no complete genome sequence of *R.anatipestifer* strain SX, the neighborhood genes of an inserted or affected gene are shown in this figure according to the full genome of type strain DSM15868 (GenBank accession No: CP002346). Red box arrows: the inserted or affected genes; Yellow box arrows: neighborhood genes; White box arrows: pseudo genes; Blue arrows: inserted sites on the genome.

**Additional file 4:** **None of the six recombinant proteins generated pores in duck erythrocyte membranes.** Cells were observed by phase contrast microscopy at 12 h post-exposure. (A) rRiean_0317; (B) rRiean_0790; (C) rRiean_0653; (D) rRiean_1027 (rOmpA1467); (E) rRiean_1143; (F) rRiean_1561. (G) the culture supernatant of the wild type SX as positive control.

## Data Availability

The data and materials in this study are available from the corresponding author upon reasonable request.

## Abbreviations

ATCC: American Type Culture Collection
BLAST: Basic Local Alignment Search Tool
COG: Clusters of Orthologous Group
dba^−^: The strains did not have hemolytic activity on duck blood agar
*E. coli*: *Escherichia coli*
HGA: Homogentisic acid
LB: Luria broth
LD_50_: Median lethal dose
NEB: New England Biolabs
OMP: Outer membrane protein
PCR: Polymerase chain reaction
*R. anatipestifer*: *Riemerella anatipestifer*
SDS-PAGE: Sodium dodecyl sulfate polyacrylamide gel electrophoresis
TSB: Tryptic soybean broth
WT: Wild type

## References

[1] Ruiz JA, Sandhu TS, Swayne DE, Glisson JR, McDougald LR, Nolan LK, Suarez DL, Nair VL (2013). *Rimerella anatipestifer* infection. Diseases of poultry.

[2] Brogden KA, Rhoades KR, Rimler RB (1982). Serologic types and physiologic characteristics of 46 avian *Pasteurella anatipestifer* cultures. Avian Dis.

[3] Hinz KH, Ryll M, Kohler B, Glunder G (1998). Phenotypic characteristics of *Riemerella anatipestifer* and similar micro-organisms from various hosts. Avian Pathol.

[4] Gong Y, Yang Y, Chen Y, Sun B, Xue Y, Xu X, Wang X, Islam N, Du X, Hu Q (2020). Characterization of the hemolytic activity of *Riemerella anatipestifer*. Microbiology.

[5] Crasta KC, Chua KL, Subramaniam S, Frey J, Loh H, Tan HM (2002). Identification and characterization of CAMP cohemolysin as a potential virulence factor of *Riemerella anatipestifer*. J Bacteriol.

[6] Kulasekara HD (2014). Transposon mutagenesis. Methods Mol Biol.

[7] Shoemaker NB, Getty C, Gardner JF, Salyers AA (1986). Tn4351 transposes in *Bacteroides* spp. and mediates the integration of plasmid R751 into the *Bacteroides* chromosome. J Bacteriol.

[8] McBride MJ, Baker SA (1996). Development of techniques to genetically manipulate members of the genera *Cytophaga*, *Flavobacterium*, *Flexibacter*, and *Sporocytophaga*. Appl Environ Microbiol.

[9] Hu Q, Zhu Y, Tu J, Yin Y, Wang X, Han X, Ding C, Zhang B, Yu S (2012). Identification of the genes involved in *Riemerella anatipestifer* biofilm formation by random transposon mutagenesis. PLoS ONE.

[10] Ni X, Jiang P, Xing L, Ou C, Yu H, Qi J, Sun B, Cui J, Wang G, Hu Q (2016). Genome-wide mining of potential virulence-associated genes in *Riemerella anatipestifer* using random transposon mutagenesis. Vet Microbiol.

[11] Xing L, Yu H, Qi J, Jiang P, Sun B, Cui J, Ou C, Chang W, Hu Q (2015). *ErmF* and *ereD* are responsible for erythromycin resistance in *Riemerella anatipestifer*. PLoS ONE.

[12] Cooper AJ, Kalinowski AP, Shoemaker NB, Salyers AA (1997). Construction and characterization of a *Bacteroides thetaiotaomicron recA* mutant: transfer of *Bacteroides* integrated conjugative elements is *RecA* independent. J Bacteriol.

[13] McBride MJ, Braun TF, Brust JL (2003). *Flavobacterium johnsoniae* GldH is a lipoprotein that is required for gliding motility and chitin utilization. J Bacteriol.

[14] Alvarez B, Secades P, Prieto M, McBride MJ, Guijarro JA (2006). A mutation in *Flavobacterium psychrophilum tlpB* inhibits gliding motility and induces biofilm formation. Appl Environ Microbiol.

[15] BLAST server. https://blast.ncbi.nlm.nih.gov/Blast.cgi. Accessed 12 July 2018

[16] PSORTb v.3.0. http://www.psort.org/. Accessed 15 Dec 2018

[17] Hu Q, Miao S, Ni X, Lu F, Yu H, Xing L, Jiang P (2013). Construction of a shuttle vector for use in *Riemerella anatipestifer*. J Microbiol Methods.

[18] Hu Q, Han X, Zhou X, Ding C, Zhu Y, Yu S (2011). OmpA is a virulence factor of *Riemerella anatipestifer*. Vet Microbiol.

[19] Xu X, Xu Y, Miao S, Jiang P, Cui J, Gong Y, Tan P, Du X, Islam N, Hu Q (2020). Evaluation of the protective immunity of *Riemerella anatipestifer* OmpA. Appl Microbiol Biotechnol.

[20] Spellerberg B, Pohl B, Haase G, Martin S, Weber-Heynemann J, Lutticken R (1999). Identification of genetic determinants for the hemolytic activity of *Streptococcus agalactiae* by ISS1 transposition. J Bacteriol.

[21] Hirono I, Lee SJ, Aoki T (1998). An accessory protein of the iron-regulated hemolysin of *Edwardsiella tarda* is necessary for hemolytic activity. Fisheries Sci.

[22] Stoebner JA, Payne SM (1988). Iron-regulated hemolysin production and utilization of heme and hemoglobin by *Vibrio cholerae*. Infect Immun.

[23] Lu F, Miao S, Tu J, Ni X, Xing L, Yu H, Pan L, Hu Q (2013). The role of TonB-dependent receptor TbdR1 in *Riemerella anatipestifer* in iron acquisition and virulence. Vet Microbiol.

[24] Conserved Domain Database. https://www.ncbi.nlm.nih.gov/Structure/cdd/cdd.shtml. Accessed 5 Aug 2020

[25] Parker AC, Bergonia HA, Seals NL, Baccanale CL, Rocha ER (2020). The *uroS* and *yifB* genes conserved among tetrapyrrole synthesizing-deficient *Bacteroidales* are involved in *Bacteroides fragilis* heme assimilation and survival in experimental intra-abdominal infection and intestinal colonization. Infect Immun.

[26] Sepulveda Cisternas I, Salazar JC, Garcia-Angulo VA (2018). Overview on the bacterial iron-riboflavin metabolic axis. Front Microbiol.

[27] Vinella D, Albrecht C, Cashel M, D’Ari R (2005). Iron limitation induces SpoT-dependent accumulation of ppGpp in *Escherichia coli*. Mol Microbiol.

[28] Knovich MA, Storey JA, Coffman LG, Torti SV, Torti FM (2009). Ferritin for the clinician. Blood Rev.

[29] Cianciotto NP (2015). An update on iron acquisition by *Legionella pneumophila*: new pathways for siderophore uptake and ferric iron reduction. Future Microbiol.

[30] Chatfield CH, Cianciotto NP (2007). The secreted pyomelanin pigment of *Legionella pneumophila* confers ferric reductase activity. Infect Immun.

[31] Mavromatis K, Lu M, Misra M, Lapidus A, Nolan M, Lucas S, Hammon N, Deshpande S, Cheng JF, Tapia R, Han C, Goodwin L, Pitluck S, Liolios K, Pagani I, Ivanova N, Mikhailova N, Pati A, Chen A, Palaniappan K, Land M, Hauser L, Jeffries CD, Detter JC, Brambilla EM, Rohde M, Goker M, Gronow S, Woyke T, Bristow J, Eisen JA, Markowitz V, Hugenholtz P, Klenk HP, Kyrpides NC (2011). Complete genome sequence of *Riemerella anatipestifer* type strain (ATCC 11845). Stand Genomic Sci.

