# Supplementary material for: Identification of genetic determinants of hemolytic activity of Riemerella anatipestifer using random transposon mutagenesis
Source: Vet Res. 2021 Feb 12;52:19. doi: 10.1186/s13567-021-00900-6 (PMC7881567; doi:10.1186/s13567-021-00900-6)
Supplement: Supplementary file 1 — Additional file 1: Strains, plasmids and primers used in this study. [file 13567_2021_900_MOESM1_ESM.doc]

**Additional file 1 Strains, plasmids and primers used in this study**

| **Strains, plasmids or primers** | **Description** | **Source or reference** |
| --- | --- | --- |
| **Strains** | | |
| SX | Hemolytic *Riemerella anatipestifer* strain, *ereD*^r^ (erythromycin resistance) | [11] |
| *Escherichia coli* S17-1 | Tp^R^ Sm^R^ recA, thi, pro, hsdR^-^M^+^RP4: 2-Tc: Mu: Km Tn7, λpir | Biomedal |
| *E. coli* BW19851 | RP4-2 tet::Mu-1kan::Tn7-integrant uidA(deltaMlu1)::pir+ recA1 creC510 hsdR17 endA1 zbf-5 thi | ATCC |
| BW19851 (pEP4351) | *E. coli* BW19851 carrying the plasmid pEP4351, chloramphenicol resistant | [13] |
| M2 (pRES-Riean_0790) | Mutant M2 was complemented with shuttle plasmid pRES-Riean_0790 | This study |
| M18(pRES-Riean_0653) | Mutant M18 was complemented with shuttle plasmid pRES-Riean_0653 | This study |
| *E. coli* BL21(DE3) | fhuA2 [lon] ompT gal (λ DE3) [dcm] ΔhsdS λ DE3 = λ sBamHIo ΔEcoRI-B int::(lacI::PlacUV5::T7 gene1) i21 Δnin5 | NEB |
| **Plasmids** | | |
| pEP4351 | pir-requiring R6K oriV; RP4 oriT; Cm^r^Tc^r^ (Em^r^); vector used for Tn4351 mutagenesis | [12] |
| pEP4351-cfxA | part of *ermF* and *tetX* in Tn4351 was replaced with cefoxitin-resistant gene *cfxA* | This study |
| pRES | *E.coli.*-*R. anatipestifer* shuttle vector |  |
| pRES-Riean_0790 | pRES containing *ompA* promoter and Riean_0790 ORF, ermF^r^ (Ap^r^) | This study |
| pRES-Riean_0653 | pRES containing *ompA* promoter and Riean_0653 ORF, ermF^r^ (Ap^r^) | This study |
| **Primers for identification of Tn4351 insertion site on the genome of strain SX** | | |
| 340 | 5'-GACTTGGATACCTCACGCC-3' | [13] |
| 341 | 5'-TTGGAAATTTTCTGGGAGG-3' | [13] |
| TN-1 | 5'-GGACCTACCTCATAGACAA-3' | [14] |
| IS4351-F | 5'-TCAGAGTGAGAGAAAGGG-3' | [14] |
| SP1 | 5'-CTCCCAGAAAATTTCCAAGACTCTCA-3' | [9] |
| SP2 | 5'-TAAAGTGCTGACCCGTAAAACGAAC-3' | [9] |
| SP3 | 5'- GTGGTAGCTATAGCATGGAGCTTGC-3' | [9] |
| **Primers for amplifying cfxA to construct pEP4351-cfxA** | | |
| cfxA P1 | ATAGGTCCTCCAAAATCAGTTCTTTAGCGATTAC (PpuMI site underlined) | This study |
| cfxA P2 | TAACTTAAGCAAATTATAATAGAAGCAATTTGATTAGTAATCTAAA (AflII site underlined) | This study |
| **Primers for construction of the complementary plasmids** | | |
| cRiean_0653 P1 | 5'- TACTCGAGATGAAAAAACTACTACTCGCAGCACT -3' (XhoI site underlined) | This study |
| cRiean_0653 P2 | 5'- CTGCATGCTTAAATCAAAATTTAGTTAAATCAAATCTAC -3' (SphI site underlined) | This study |
| cRiean_0790 P1 | 5'-TACTCGAGATGATTAAGAAATTATCATTAGTCTCTATGTTCT-3' (XhoI site underlined) | This study |
| cRiean_0790 P2 | 5'-GCAGCATGCTTAAATATTAAAATTTAGAATCTTACTTGTAG-3' (SphI site underlined) | This study |
| **Primers for amplifying gene ORFs to construct the recombinant plasmids to express in *E. coli*** | | |
| Riean_1561 P1 | 5'- TAGGATCCATGAAAACAAAATTACTATTAACTCTT-3' (BamHI site underlined) | This study |
| Riean_1561 P2 | 5'- TACTCGAGTTAAAACCCTACATTAAGACCAA-3'(XhoI site underlined) | This study |
| Riean_1143 P1 | 5'- ATGGATCCATGAAAAAATATTTTTTTCTATCATTATCGCT-3' (BamHI site underlined) | This study |
| Riean_1143 P2 | 5'- GGTACTCGAGCTATTTTACTTCTAAAAAGTTATCTTCTGGATTT-3'(XhoI site underlined) | This study |
| Riean_0790 P1 | 5'- TAGGATCCATGATTAAGAAATTATCATTAGTCTCTATGTT-3' (BamHI site underlined) | This study |
| Riean_0790 P2 | 5'- GTCTCGAGTTAGAATCTTACTTGTAGATTGATAAAATAGTTT-3'(XhoI site underlined) | This study |
| Riean_0653 P1 | 5'- TAGGATCCATGAGCTGCACAGACAGAAACA-3' (BamHI site underlined) | This study |
| Riean_0653 P2 | 5'- CGCTACTCGAGTCAAAATTTAGTTAAATCAAATCTACCT-3'(XhoI site underlined) | This study |
| Riean_0317 P1 | 5'- ATGGATCCATGAAAAAAGTTATTTTAAGTATTGCT-3' (BamHI site underlined) | This study |
| Riean_0317 P2 | 5'- TACTCGAGTTATTTTTTGAGACTATCTTCTACAGCC-3'(XhoI site underlined) | This study |
