# Supplementary figures and images for: Identification of genetic determinants of hemolytic activity of Riemerella anatipestifer using random transposon mutagenesis
Source: Vet Res. 2021 Feb 12;52:19. doi: 10.1186/s13567-021-00900-6 (PMC7881567; doi:10.1186/s13567-021-00900-6)

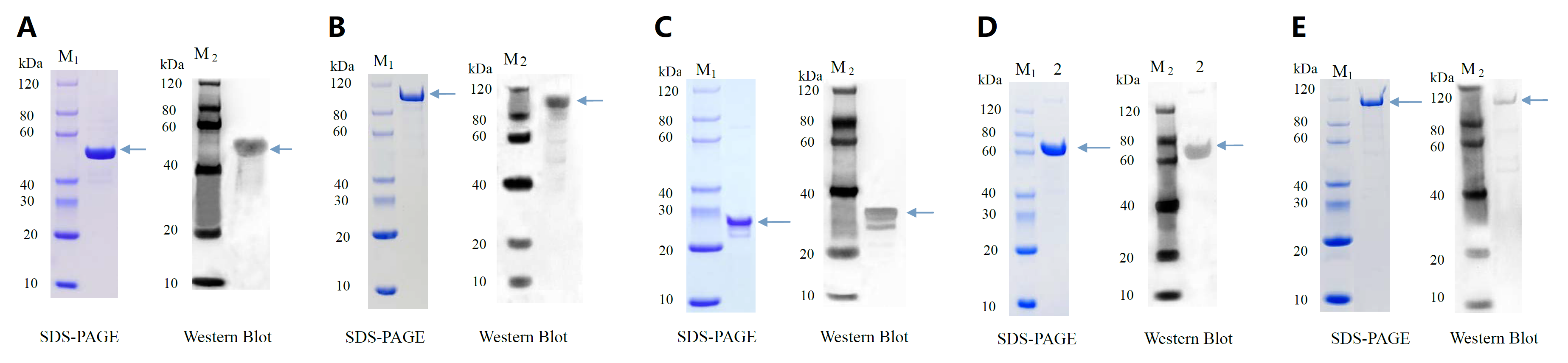

Supplement: Supplementary file 2 — Additional file 2: Detection of purified recombinant proteins by SDS-PAGE and Western blot analysis. After purification, the recombinant proteins were separated by 12% SDS-PAGE under reducing conditions. Gels were either stained with Coomassie blue or electroblotted onto a polyvinylidene fluoride membrane. Mouse anti-His tag antibody was used as the primary antibody for Western blot analysis. M1: protein marker for SDS-PAGE. M2: protein marker for Western blot analysis. (A) Detection of purified rRiean_0317 by SDS-PAGE and Western blot analysis. (B) Detection of purified rRiean_0373 by SDS-PAGE and Western blot analysis. (C) Detection of purified rRiean_0653 by SDS-PAGE and Western blot analysis. (D) Detection of purified rRiean_1143 by SDS-PAGE and Western blot analysis. (E) Detection of purified rRiean_1561 by SDS-PAGE and Western blot analysis. [file 13567_2021_900_MOESM2_ESM.tif]

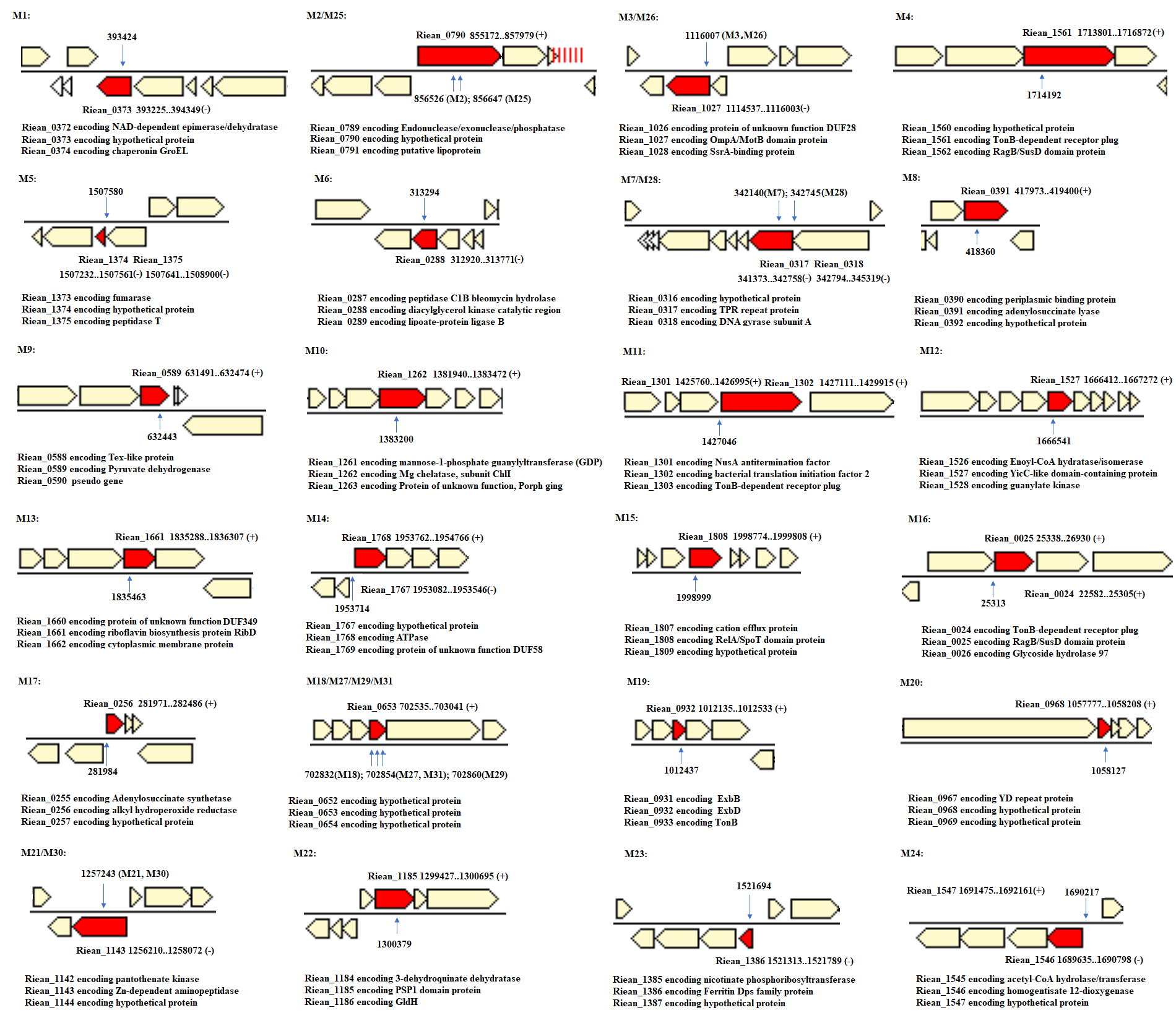

Supplement: Supplementary file 3 — Additional file 3: The Location of Tn4351 insertions on the genome of hemolytic-defective mutants. Since there is no complete genome sequence of R.anatipestifer strain SX, the neighborhood genes of an inserted or affected gene are shown in this figure according to the full genome of type strain DSM15868 (GenBank accession No: CP002346). Red box arrows: the inserted or affected genes; Yellow box arrows: neighborhood genes; White box arrows: pseudo genes; Blue arrows: inserted sites on the genome. [file 13567_2021_900_MOESM3_ESM.tif]

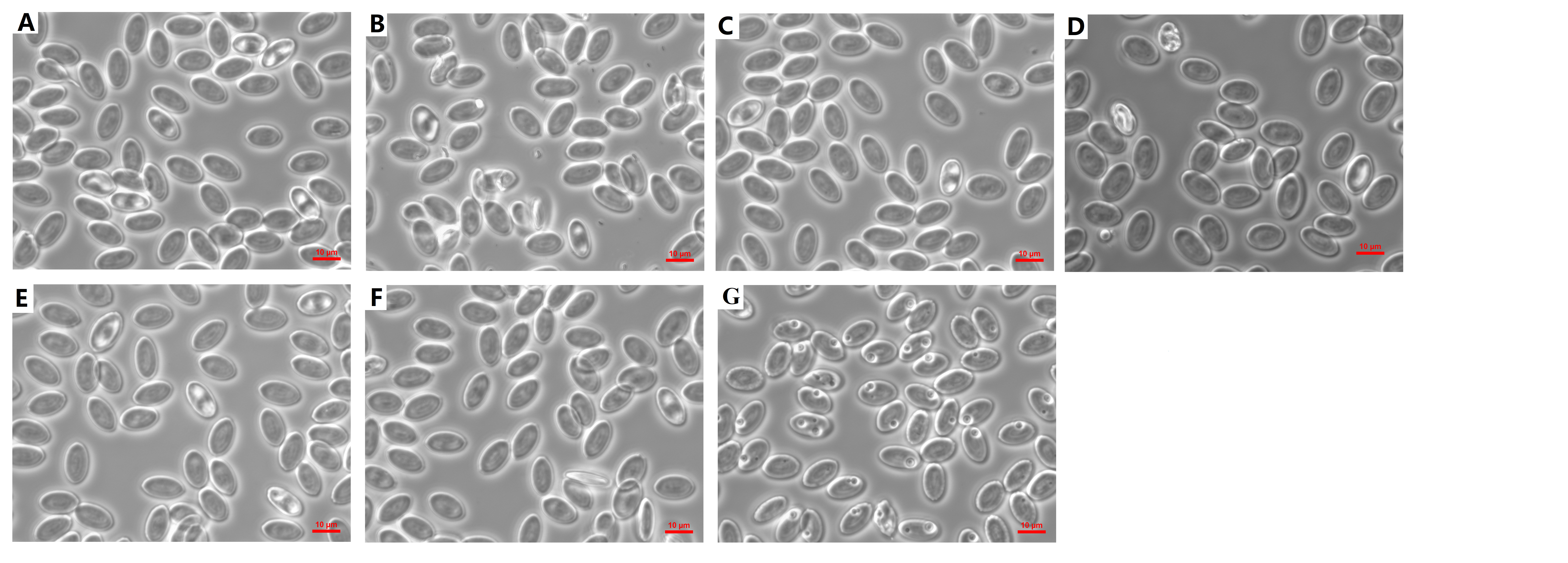

Supplement: Supplementary file 4 — Additional file 4: None of the six recombinant proteins generated pores in duck erythrocyte membranes. Cells were observed by phase contrast microscopy at 12 h post-exposure. (A) rRiean_0317; (B) rRiean_0790; (C) rRiean_0653; (D) rRiean_1027 (rOmpA1467); (E) rRiean_1143; (F) rRiean_1561. (G) the culture supernatant of the wild type SX as positive control. [file 13567_2021_900_MOESM4_ESM.jpg]
